# Editorial: Terahertz technologies for biosensing and biomedical analysis

**DOI:** 10.3389/fbioe.2023.1268427

**Published:** 2023-08-21

**Authors:** Yiming Zhu

**Affiliations:** Terahertz Technology Innovation Research Institute, Terahertz Spectrum and Imaging Technology Cooperative Innovation Center, Shanghai Key Lab of Modern Optical System, University of Shanghai for Science and Technology, Shanghai, China

**Keywords:** terahertz technology, biomedicine, detection, neuroscience, spectral database, instrumentation

Terahertz technology mainly refers to electromagnetic radiation technology with a frequency between 0.1 and 10 THz, which is in the transition region between macro electronics and micro photonics. This frequency band contains important information such as spatial conformation, which directly represents the function of biological macromolecules, which cannot be detected in other electromagnetic wave segments. In the field of biomedicine microcosmic, terahertz technology will provide revolutionary scientific methods for revealing the material laws of interaction between biological macromolecules and cells, presenting the physical characteristics of these effects and activities, and finally explaining various life phenomena. At the macro level of biomedicine, it will bring revolutionary changes to the diagnosis, treatment, evaluation, monitoring and early warning of diseases and subsequent drug design, research and development, production and evaluation.

First, terahertz technology is used to study biomolecules and cells. Zhang et al. the myristoylphosphatidycholine in water (DMPC) bilayer of atomic and molecular dynamics simulation on carefully layered molecular numerical calculation and calibration of optical dielectric constant, The spatially resolved (subnanometer) dielectric spectra of phospholipid bilayers over a wide range from terahertz to mid-infrared have been demonstrated for the first time. Zhang et al. used terahertz spectroscopy to obtain refractive indices of three cell types, *E. coli*, stem cells and cancer cells, and their states under stress. Terahertz spectroscopy also has potential applications for detecting protein concentrations in urine samples. Xue et al. were able to distinguish proteins of different molecular weights by analyzing terahertz spectral absorption in the 0.5–1.2 terahertz band. At the same concentration, the terahertz absorption of high molecular weight protein is greater than that of low molecular weight protein. It is found that terahertz time-domain spectroscopy technology utilizes the vibration and interaction between electromagnetic waves and substances in the terahertz frequency range, and obtains the spectral information of samples by measuring the propagation time and amplitude of light waves, so as to realize substance analysis and detection.

Second, terahertz waves are useful in medicine for the detection and diagnosis of diseases. For example, Shi et al. using a high-resolution terahertz imaging system, can scan non-tumor adjacent tissue and gastric cancer tissue sections by observing the difference in transmittance between different tissue layers in the terahertz image, i.e., the submucosa has lower transmittance than the mucosal and muscle layers of non-tumor adjacent tissue. It can realize the identification of non-tumor tissue and tumor tissue. Li et al. identified and confirmed the types of gout stones in a timely manner by combining Raman spectroscopy and terahertz spectroscopy techniques to reduce pain and inflammation in patients and prevent complications associated with gout stones. Then, terahertz waves are widely used in the field of neuroscience, and research has simulated and experimented with the terahertz interactions of nerve fibers and neurons to explore their electromagnetic and transport properties. Guo et al. investigated the electromagnetic properties of nerve fibers at THz-FIR band. Firstly, the electromagnetic transmission model of nerve fibers is established and theoretical research is carried out. Secondly, a pattern matching algorithm named RNMMA is proposed to calculate the transmission characteristics of terahertz FIR waves at Ranvier nodes. By comparing with the results of electromagnetic simulation software, the scattering matrix obtained by this algorithm is in good agreement with the actual situation. The study reveals how terahertz FIR signals can be transmitted forward through Ranvier nodes with low losses. Then, Shaoqing et al. established a propagation and thermal effect model of the interaction between 0.3 and 3 THz waves and neurons, and took field intensity and temperature changes as evaluation criteria. The effect of cumulative radiation of terahertz waves on neuronal structure was investigated experimentally. The results show that the frequency and power of terahertz waves are the main factors affecting the neuronal field intensity and temperature, and there is a positive correlation between them.

In addition, Huang et al. introduced the research achievements of terahertz time-domain spectroscopy in the analysis of chemical drugs, traditional Chinese medicine and biological drugs in recent 10 years. The scientific feasibility of terahertz time-domain spectroscopy in drug detection is also revealed. The problems existing in the practical application of terahertz time-domain spectroscopy and its further development prospects in drug analysis are discussed. It shows that terahertz technology is widely used in the analysis of chemical drugs, traditional Chinese medicine and biological drugs, and can be used to study the structure and interaction of drugs, identify the authenticity of drugs, whether the deterioration.

Finally, the combination of terahertz technology and other technologies can be used for biomedical database construction, instrument development, etc., which promotes its development in the biomedical field. Li et al. established a spectral database of gout stones and urinary stones by combining Raman spectroscopy and terahertz spectroscopy techniques. The database will provide accurate and comprehensive technical support for the rapid diagnosis of clinical gout. Biosensing is developed through the combination of terahertz spectroscopy and microfluidic technology, which can be used for biological sample detection. On the one hand, Zhang et al. constructed an automated, high-throughput sample preparation and detection system for inquiring biological cell phenotypes to measure the refractive index of living cells in near-physiological environments, which is suitable for cell health assessment and drug discovery. On the other hand, Fu et al. investigated the structure of a polydimethylsiloxane microfluidic chip (M-chip) suitable for measuring water-based samples, in particular the effect of the cavity depth of the M-chip on the terahertz spectrum. When the depth is less than 210 μm, the Fresnel formula of the two-interface model should be used for the analysis of the terahertz spectral data, and when the depth is not less than 210 μm, the Fresnel formula of the single-interface model can be used. Terahertz wave construction practical instrument. Wang et al. built a terahertz attenuated total reflection spectrometer with a signal-to-noise ratio (SNR) of 40–60 dB. The device can be used to detect liquid biological samples with high sensitivity, such as the identification and quantitative analysis of lactose aqueous solution.

In short, terahertz technology has a wide range of application prospects in all aspects of the biomedical field, although there are still some technical challenges and problems to overcome, but with the continuous development of technology and in-depth research, terahertz technology will play a greater role in the future.

